# Silica-Based and Borate-Based, Titania-Containing Bioactive Coatings Characterization: Critical Strain Energy Release Rate, Residual Stresses, Hardness, and Thermal Expansion

**DOI:** 10.3390/jfb7040032

**Published:** 2016-12-01

**Authors:** Omar Rodriguez, Ali Matinmanesh, Sunjeev Phull, Emil H. Schemitsch, Paul Zalzal, Owen M. Clarkin, Marcello Papini, Mark R. Towler

**Affiliations:** 1 Department of Mechanical & Industrial Engineering, Ryerson University, Toronto, ON M5B 2K3, Canada; amatinma@ryerson.ca (A.M.); sunjeev.phull@ryerson.ca (S.P.); mpapini@ryerson.ca (M.P.); mtowler@ryerson.ca (M.R.T.); 2 St. Michael’s Hospital, Toronto, ON M5B 1W8, Canada; SchemitschE@smh.ca; 3 Department of Surgery, University of Western Ontario, London, ON N6A 4V2, Canada; 4 Oakville Trafalgar Memorial Hospital, Oakville, ON L6J 3L7, Canada; paulzalzal@gmail.com; 5 Faculty of Health Sciences, Department of Surgery, McMaster University, Hamilton, ON L8S 4L8, Canada; 6 School of Mechanical and Manufacturing Engineering, Dublin City University, Dublin D09 W6Y4, Ireland; owen.clarkin@dcu.ie; 7 Department of Biomedical Engineering, University of Malaya, Kuala Lumpur 50603, Malaysia

**Keywords:** enameling, coefficient of thermal expansion, borate-based glass, indentation

## Abstract

Silica-based and borate-based glass series, with increasing amounts of TiO_2_ incorporated, are characterized in terms of their mechanical properties relevant to their use as metallic coating materials. It is observed that borate-based glasses exhibit CTE (Coefficient of Thermal Expansion) closer to the substrate’s (Ti6Al4V) CTE, translating into higher mode I critical strain energy release rates of glasses and compressive residual stresses and strains at the coating/substrate interface, outperforming the silica-based glasses counterparts. An increase in the content of TiO_2_ in the glasses results in an increase in the mode I critical strain energy release rate for both the bulk glass and for the coating/substrate system, proving that the addition of TiO_2_ to the glass structure enhances its toughness, while decreasing its bulk hardness. Borate-based glass BRT3, with 15 mol % TiO_2_ incorporated, exhibits superior properties overall compared to the other proposed glasses in this work, as well as 45S5 Bioglass^®^ and Pyrex.

## 1. Introduction

Direct skeletal attachment (DSA) is a method used in prosthetics in which a metallic implant is attached directly to the patient’s bone at the residual limb; concerns regarding DSA include infection and skin irritation [1,2,3]. Different approaches have been taken towards re-designing DSA devices for improving patient outcomes; these approaches usually involve modification of the surface by sandblasting the device surface, titanium plasma-spraying, plasma-spraying with hydroxyapatite (HA), coating the implant with a titanium dioxide (TiO_2_) layer through anodic oxidation, and applying a coating made from bioactive glass [4,5,6]. From these approaches, bioactive glasses have shown encouraging results over these other technologies when used as coatings [5].

The potential of bioactive glasses as coatings was first postulated with the development of Hench’s 45S5 Bioglass^®^ in the 1960s [7]. Bioglass^®^ was the first synthetic material to chemically adhere to both hard and soft tissue [7]. Although bioactive glasses have been employed for coating metals [8,9,10,11], these compositions have all, to date, contained alumina [8,11], which is associated with both defective bone mineralization and neurotoxicity [12]. Other compositions have been deficient in zinc [9,10,11], an antibacterial component [13,14,15] which aids the healing process by inhibiting the growth of caries-related bacteria such as *Streptococcus mutans* [16].

This work considers two distinct glass series, one based on silica (SiO_2_), one based on borate (B_2_O_3_), with increasing amounts of titanium dioxide (TiO_2_) incorporated at the expense of silica and borate, respectively. B_2_O_3_ has been shown to reduce the coefficient of thermal expansion (CTE) of glasses [17], so that borate glasses have CTEs closer to that of the metallic substrate to be coated (typically Ti6Al4V, with a CTE of 9.5 × 10^−6^/°C in the range of 0–315 °C [18]). Processing such glasses for use as coatings (e.g., through enameling [9], plasma spraying [19], electrophoretic deposition [20,21], or glazing [22]) requires heat treatment to allow for the glass to react with the substrate surface thus creating a chemical bond [23,24]. Once the bond has formed and the assembly is cooled, a difference in CTE between the glass and metal will induce residual stresses, hence causing cracks to appear in the glass or at the glass/substrate interface. For this reason, a borate-based glass series is proposed, to evaluate the effect of B_2_O_3_ on its coating capability by means of its reduced CTE compared to silica-based glasses; a silica-based glass series is also proposed, with a homologous composition to that of the borate-based glass series, to allow for the evaluation of the effect of B_2_O_3_ versus SiO_2_ on the resultant properties of the coating. Additionally, TiO_2_ is incorporated in these glasses as it helps promote a more stable chemical bond when coating such a glass onto Ti6Al4V [17]. Additionally, titanium is known to create a permanent bond to bone, via osseointegration [25,26].

In terms of metal coating techniques, enameling is probably one of the most widely used. Among the different findings in the literature, researchers have found that low firing temperatures and short sintering times did not help the glass to spread along the substrate surface, ultimately resulting in a very porous coating [8], that silica-based glasses with significantly high percentage of SiO_2_ exhibit better adhesion to the metallic substrate due to lesser thermal expansion mismatch between the glass and the substrate [27], and that smaller processing windows (the range between glass transition and crystallization temperature where the coating is heat treated to) favors crystallization hence reducing bioactivity of the glass [28]. Other coating methods have been explored, including reactive plasma spraying [19], electrophoretic deposition [20], and dip coating [29]. Good adhesion has been reported using these methods; however, these methods may require sintering at high temperatures [19,20,29], which may hinder the bioactive performance of glass due to crystallization, and they produce fragile coatings [29], compromising the mechanical stability of the coating.

Indentation-based measurement methods allow a quick and qualitative measurement of the adhesion [11,30,31,32]. Such tests can also be used to quantify fracture toughness of the material by the direct measurements of cracking after indentation [33,34,35,36,37,38,39]. A common example is the Vickers indentation fracture (VIF) test, which measures the lengths of the cracks emanating from the Vickers indents. This technique was first developed by Lawn et al. [33], under the assumption that such cracks were created due to tensile stresses that form during unloading. Anstis et al. [34] validated Lawn’s model for several ceramics and glasses by comparing the fracture toughness obtained from the VIF test with the ones obtained from standard fracture tests. Later, Laugier [39] showed that indentation crack geometry in glasses and ceramics were different and claimed that Lawn’s model required some modifications when used for the evaluation of ceramic toughness, and therefore developed a new model that described the indentation cracking in ceramics more realistically.

In this study, both glass series will be evaluated to determine their coefficient of thermal expansion (CTE) and its effect on the residual stresses and strains post-coating, their critical strain energy release rate in mode I (opening) of the coating/substrate system through double-cantilever beam (DCB) specimens and of the bulk glass through Vickers indentation, and their bulk hardness.

## 2. Results

### 2.1. Coefficient of Thermal Expansion (CTE)

**Figure 1:**
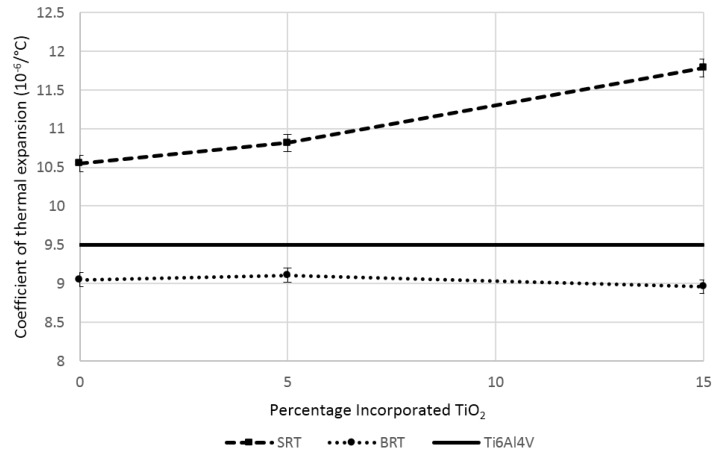
CTE for the SRT and BRT glasses, plotted along with the CTE of Ti6Al4V as a reference. Scatter bars indicate one standard deviation from the mean.

Results from the measurement of the CTE are found in Figure 1 for the SRT (silica-based) and BRT (borate-based) glass series. The CTE of the silica-based glasses were found to be consistently greater than the CTE of Ti6Al4V, with the percentage difference ranging between 11.1% and 24%, whereas the CTE for the borate-based glasses were found to be below the CTE of Ti6Al4V with a smaller percentage difference, ranging between 4.1% and 5.8%. In statistical terms, the CTE for all borate-based glasses were found to be equivalent; the CTE for SRT0 and SRT1 (0 and 5 mol% incorporated TiO_2_) were also found to be equivalent.

### 2.2. Residual Stress and Strain Analysis

**Figure 2:**
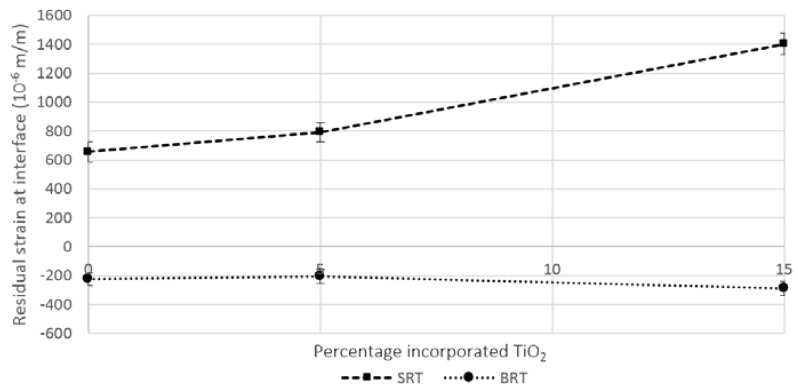
Residual strain at the substrate/coating interface using the SRT and BRT glasses as coatings. Scatter bars indicate one standard deviation from the mean.

**Figure 3:**
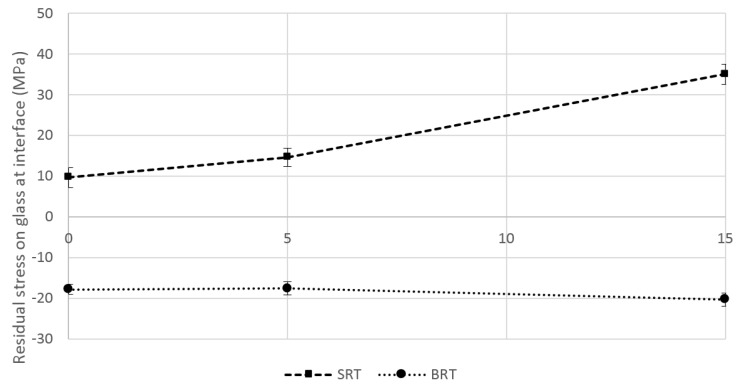
Residual stresses experienced in the glass coating at the coating/substrate interface using the SRT and BRT glasses as coatings. Scatter bars indicate one standard deviation from the mean.

Residual strain results (Equation (3)) are shown in Figure 2, where it can be seen that, in terms of the magnitude, greater strains were experienced when the silica-based coatings were employed, opposed to borate-based ones. This is due to higher CTE mismatch between the silica-based glasses and the titanium substrate, especially for the case of SRT3 (15 mol % incorporated TiO_2_). Residual stress in the glass coatings (Figure 3) at the interfacial site (Equation (4)) were found to follow a similar trend as the residual strains, with borate-based glasses exhibiting compressive residual stresses and silica-based glasses exhibiting tensile residual stresses.

### 2.3. Vickers Hardness

**Figure 4:**
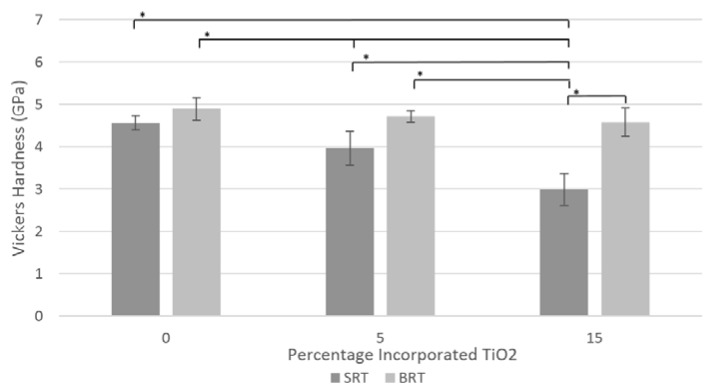
Vickers hardness for the SRT and BRT glasses. Scatter bars indicate one standard deviation from the mean. Stars and bars show statistical significance (*p* < 0.05).

A substitution of SiO_2_ for B_2_O_3_ resulted in an increase in the Vickers hardness of the glass, as shown in Figure 4, statistically significant at 5 and 15 mol % of incorporated TiO_2_. The incorporation of TiO_2_, however, did not significantly affect the hardness for the BRT glass series; for the SRT glass series, the addition of TiO_2_ at 5 mol % decreased the hardness, but further addition did not significantly decrease it.

### 2.4. Bulk Mode I Critical Strain Energy Release Rate Using Vickers Indentation

**Figure 5:**
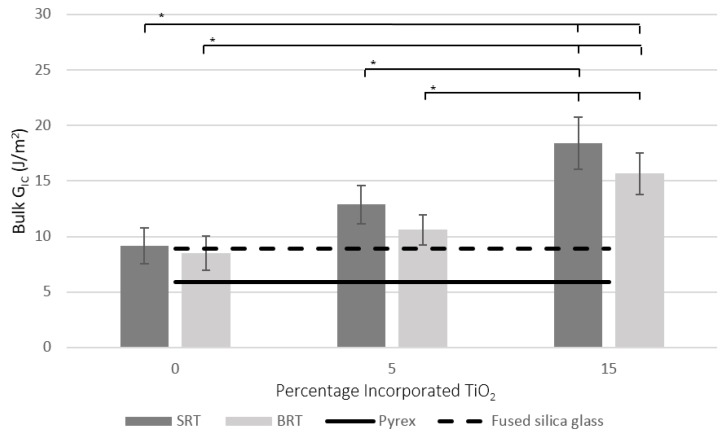
Bulk Mode I critical strain energy release rates for the SRT and BRT glasses. The G_IC_ values for fused silica glass and Pyrex obtained from the literature [40,41] are also shown for reference. Scatter bars indicate one standard deviation from the mean. Stars and bars show statistical significance (*p* < 0.05).

**Figure 6:**
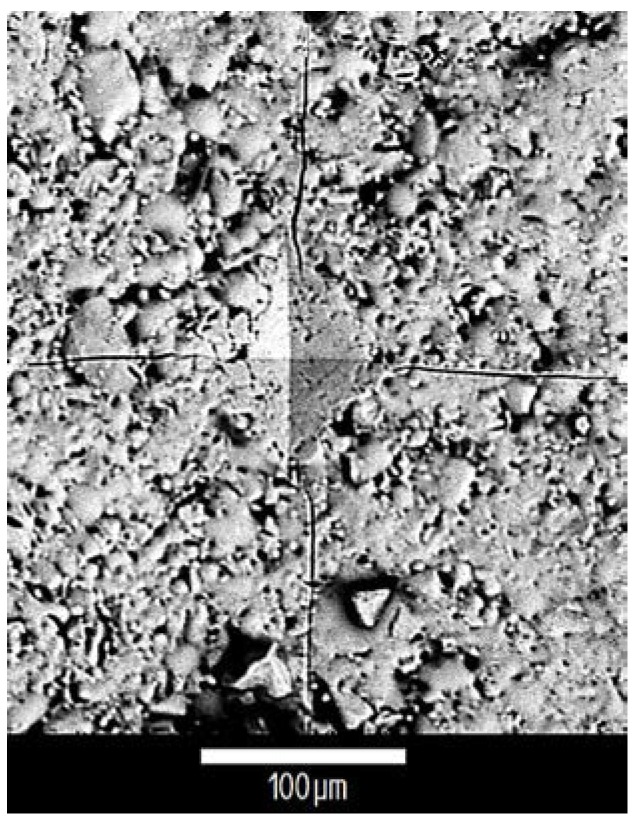
SEM of a Vickers indent on SRT0 with the emanating cracks. The average half diameter and crack length are 54.8 μm and 187.9 μm, respectively.

The Vickers indentation test was performed to measure the bulk mode I critical strain energy release rate (Equation (6)), and the results are shown in Figure 5. A sample of the SEM image for SRT0 showing the indent and the cracks emanating from it is presented in Figure 6. Figure 5 also presents as reference points the data obtained from the literature for the mode I critical strain energy release rate of fused silica based glass (99.995% SiO_2_) and Pyrex (heat resistant borosilicate glass). Based on previous studies, the fracture toughness K_IC_ and the modulus of elasticity E_c_ of fused silica glass and Pyrex are 0.80 MPa·m^1/2^ [40] and 0.63 MPa·m^1/2^ [41], and 72 GPa [40] and 67 GPa [42], respectively. The following equation [43], valid for the plane stress condition, was used to convert these K_IC_ and E_c_ values to the G_IC_ in Figure 5, yielding 8.9 J/m^2^ for fused silica and 5.9 J/m^2^ for Pyrex:

(1) $$G_{\text{IC}}=\frac{K_{\text{IC}}^{2}}{E_{c}}$$

### 2.5. Coating/Substrate System Mode I Critical Strain Energy Release Rate

**Figure 7:**
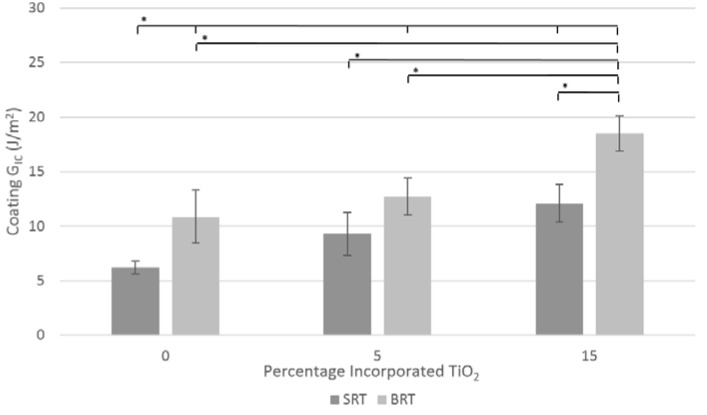
Mode I critical strain energy release rates for the coating/substrate systems with SRT and BRT glasses. Scatter bars indicate one standard deviation from the mean. Stars and bars show statistical significance (*p* < 0.05).

The mode I critical strain energy release rates for the coating/substrate system for both glass series are shown in Figure 7. Systems made with borate-based glasses exhibited higher critical strain energy release rates in mode I opposed to the silica-based coatings, with the exception of SRT1 and BRT1 (5 mol % incorporated TiO_2_), which were statistically equivalent (*p* < 0.05). As a function of the percentage of TiO_2_ incorporated, for the silica-based series, there is no significant difference in the critical strain energy release rate between 0 and 5 mol % incorporated TiO_2_ and between 5 and 15 mol % incorporated TiO_2_; however, a statistical difference is observed between 0 and 15 mol % incorporated TiO_2_. Similarly, for systems made with the borate-based series, there is no significant increase (*p* < 0.05) in the critical strain energy release rate between 0 and 5 mol % incorporated TiO_2_, whereas a significant increase is found between 0 and 15 mol % incorporated TiO_2_, and between 5 and 15 mol % incorporated TiO_2_.

## 3. Discussion

The CTE results confirmed that the borate-based glasses possessed CTEs comparable to that of Ti6Al4V, evidenced by the reduced percentage difference between the borate-based glasses and Ti6Al4V, compared to the silica-based glasses. However, the CTE for the proposed silica-based glasses was significantly lower than what has been reported for other similar glasses (e.g., CTE of Bioglass^®^ 45S5 has been reported to be 15.1 × 10^−6^/°C over the range from 200 to 400 °C [32]), indicating that the proposed silicate formulations would provide better adhesion to the metallic substrate compared to other silica-based glasses. Since the addition of TiO_2_ increased the CTE, the control silicate formulation should be analyzed to understand the causes behind the reduced CTE. Compared to 45S5, SRT0 contains a higher molar percentage of SiO_2_ and contains ZnO at 16 mol %, whereas 45S5 does not include ZnO. Higher SiO_2_ has been determined to decrease the CTE of silica-based glasses [27], which additionally explains how substituting SiO_2_ for TiO_2_ in the SRT glasses translated into an increase in the CTE. Furthermore, ZnO increase (or inclusion, in this case) has been proven to decrease the CTE of silica-based glasses [44], explaining the reduced CTE of SRT0 compared to 45S5. 

Since the CTEs of the borate-based glasses are lower than that of the substrate, the difference in shrinkage caused the glass coating to experience compressive residual stresses. Compressive residual stresses are beneficial, acting to prevent cracks from propagating, thus requiring higher stresses to cause coating failure [45,46,47]. On the other hand, the CTE of the silica-based glasses induced positive residual stresses, promoting the growth of cracks under loading [45,48].

As shown in Figure 5, the measured bulk G_IC_ of the control glass of the silica-based series, SRT0, was found to be comparable to that of fused silica glass. However, as the percentage of TiO_2_ in the silica-based glass series was increased, the mean G_IC_ value increased, with significant differences (*p* < 0.05) observed between the SRT0 and SRT3 (15 mol % incorporated TiO_2_). There is limited literature available on the mode I critical strain energy release rate (or fracture toughness) of bioactive glasses, specifically on the effect of the inclusion of TiO_2_ at the expense of the backbone component. The authors hypothesize that the observed increase in the bulk G_IC_ as the amount of TiO_2_ increased can be attributed to the presence of Ti^4+^ ions. Such ions have been shown to strengthen glass systems and to improve their mechanical properties [49], due to their small ionic radius and high electrical charge [50] that tends to strengthen the bonds in the glass. The bulk G_IC_ of the control glass in the borate series, BRT0, was found not to be significantly different (*p* < 0.05) from that of SRT0; however, the bulk G_IC_ of the remaining borate-based glasses were lower than their counterpart in the silica-based series. This is not surprising, as previous studies on boro-silicate glasses have shown that the incorporation of B_2_O_3_ at the expense of SiO_2_ can decrease the fracture toughness (directly related to G_IC_ through Equation (1)) [51,52]. Furthermore, similar to the silica-based series, the incorporation of TiO_2_ in the borate series glasses increased the fracture toughness.

In terms of the G_IC_ of the coating/substrate system, the presently used DCB testing method was first published by Matinmanesh et al. [53], establishing the G_IC_ for SRT0-based systems at 6.20 ± 0.60 J/m^2^. The current work expanded on these findings, determining that an increase in TiO_2_ content resulted in an increase in critical strain energy release rate, peaking at 12.08 ± 1.72 J/m^2^ for the silica-based coatings, and 18.50 ± 1.60 J/m^2^ for the borate-based coatings. This work supports the previous findings that titanium in the glass coating enhances chemical bonding to the titanium substrate [17], resulting in a larger measured G_IC_ for the SRT3 and BRT3 systems, both with 15 mol % of TiO_2_ incorporated. Additionally, the trends of the G_IC_ for the bulk glass and the coating/substrate system are similar, i.e., an increase in incorporated TiO_2_ translated into an increase in G_IC_. This is similar to the work of Li et al. [54], who studied the effect of the incorporation of strontium oxide into borate-based glass coatings applied to Ti6Al4V substrates, and found that G_IC_ increased as the amount of strontium oxide increased.

The residual stress in the SRT0 coating using the measured values of the CTE was estimated to be 9.6 ± 2.4 MPa (Section 2.2). This is consistent (no significant difference, *p* < 0.05) with the 8.6 ± 1.0 MPa value found by Matinmanesh et al. [53] for the same SRT0 coating/substrate system using measurements of the radius of curvature of the coated assembly.

Comparing the presented G_IC_ values in Figure 5 with those in Figure 7 revealed that the glasses in the silica-based series have higher critical strain energy release rates in bulk form compared to when they are applied to the coating/substrate. On the contrary, in the borate based series, the G_IC_ was higher for the coating/substrate system. This effect may be attributed to the nature of the residual stresses that are created during the coating process, i.e., tensile in silica-based systems and compressive in borate-based systems (Section 2.2), with compressive residual stresses providing additional resistance to crack growth.

The bulk hardness of the silica-based glass series decreased as the amount of TiO_2_ incorporated into the glasses increased, while, in statistical terms, the hardness of the borate-based glasses did not significantly change (*p* < 0.05) with the addition of TiO_2_. The hardness of the borate-based glasses however, was significantly higher than the silica-based equivalent glasses at 5 and 15 mol % incorporated TiO_2_. The decrease in hardness with increasing incorporation of TiO_2_ is consistent with the observed increase in fracture toughness, which is inversely proportionally to the hardness of the glass (Equation (6)).

## 4. Materials and Methods

### 4.1. Glass Preparation

Silica-based and borate-based glasses in this study were synthesized (compositions and nomenclature are reported in Table 1) and characterized by X-ray diffraction (XRD), differential scanning calorimetry (DSC), and Fourier transform infrared spectroscopy (FTIR), among other techniques [55]. TiO_2_ was added at the expense of SiO_2_ for the SRT series and at the expense of B_2_O_3_ for the BRT series. The glasses were prepared by weighing out appropriate amounts of analytical grade reagents (Fisher Scientific, Ottawa, ON, Canada & Sigma-Aldrich, Oakville, ON, Canada), firing in silica crucibles (1400–1500 °C for 1 h for the silica-based glasses, 1200 °C for 1 h for borate-based glasses), and shock quenching in water. The resulting frit was then ball-milled, and sieved to retrieve glass particulates ≤20 μm.

#### 4.1.1. Discs Preparation

Approximately 200 mg of each glass were pressed into a cylindrical mold with the diameter of 6 mm using a hydraulic press with pressure ranging between 2500 and 3000 psi. The sample thicknesses were 2.84 ± 0.19 mm. The pressed discs were then heat treated to promote the coalescence of glass particles and create a sturdy solid to be used for CTE and hardness testing. The heat treatment consisted of firing the discs at temperature T_coat_ (Table 2) for 15 min, then allowing them to cool down to room temperature. 

#### 4.1.2. Coating Preparation

Coatings were prepared following the procedure developed by Matinmanesh et al. [53] for enameling Ti6Al4V with bioactive glasses in an ethanol-based suspension. Ti6Al4V substrate samples were degreased and cleaned in ethanol prior to coating. For each glass formulation, a suspension of the glass powder in ethanol (ratio of 5:1, ethanol to glass mass) was deposited on the substrates. Afterwards, the coatings were allowed to air-dry for 30 min, and were then fired at temperature T_coat_, ranging between the glass transition temperature (T_g_) and the crystallization temperature (T_*x*_) of each glass (Table 2), for 15 min.

### 4.2. Coefficient of Thermal Expansion (CTE) Measurement by Linear Dilatometry

The CTE of each glass was tested based on the current ASTM E228 “Standard Test Method for Linear Thermal Expansion of Solid Materials with a Push-Rod Dilatometer” [56]. Samples were prepared following the procedure outlined in Section 4.1.1, with samples measuring 6 mm in diameter and 12 mm in height (by stacking 4 discs), and tested with a Netzsch DIL 402 PC dilatometer (Netzsch Instruments, Burlington, MA, USA). A heating rate of 4 °C/min was employed, with a testing temperature range from 25 to 300 °C for both the glass series. Based on the measured lengths and temperature changes, CTE was determined as

(2) $${\left[\alpha_{m}\right]}_{Ti}={\left[\frac{1}{\Delta T}\frac{\Delta L}{L_{0}}\right]}_{Ti}$$

where *α_m_* is the mean CTE of the glass, *ΔT* is the change in temperature with respect to the initial temperature, *L*_0_ is the initial length of the test specimen, and *ΔL* is the change in length of the sample with respect to the initial length *L*_0_.

### 4.3. Residual Stress and Strain Analysis

Due to the mismatch between the CTE of the substrate and of the coating, residual stresses at the interface were induced. Based on the measured CTE as per Section 4.2, and based on the cooling profile and temperatures as per Section 4.1.2, residual strains were computed as

(3) $$\varepsilon_{\text{res}}=\left(\alpha_{\text{glass}}-\alpha_{\text{Ti}6\text{Al}4V}\right)\left(T_{\text{coat}}-T_{i}\right)$$

where ε_res_ is the calculated residual strain, α_glass_ is the CTE of the glass, α_Ti6Al4V_ is the CTE of the titanium substrate (9.5 × 10^−6^/°C), T_coat_ is the coating temperature (from Table 2), and T_i_ is the room temperature (25 °C). This approach has already been proposed and verified by Oel and Frechette [57]. To determine the residual stresses, Yu et al. [58] proposed the use of beam theory on bi-layer materials with different CTEs subjected to thermal loading. The residual stress at the interface experienced by the glass coating is

(4) $$\sigma_{\text{res}\_\text{glass}}=\frac{P\left(t_{s}+t_{c}\right)\left(\frac{t_{c}}{2}\right)}{2I_{c}+\frac{2E_{s}I_{s}}{E_{c}}}$$

where

(5) $$P=\frac{\varepsilon_{\text{res}}}{\frac{1}{E_{c}A_{c}}+\frac{1}{E_{s}A_{s}}+\frac{{\left(t_{s}+t_{c}\right)}^{2}}{4\left(E_{c}I_{c}+E_{s}I_{s}\right)}}$$

and t_s_ is the thickness of the titanium substrate (3.15 mm), t_c_ is the thickness of the glass coating (90 µm), I_s_ is the second moment of area of the titanium substrate, defined as $I_{s}=\frac{{w\;t}_{s}^{3}}{12}$ where w is the width of the titanium substrate and of the glass coating (11 mm), I_c_ is the second moment of area of the glass coating, defined as $I_{c}=\frac{{w\;t}_{c}^{3}}{12}$ E_s_ is the modulus of elasticity of the titanium substrate (110 GPa), E_c_ is the modulus of elasticity of the glass coating (35 GPa [59]), A_s_ is the cross-sectional area of the titanium substrate, defined as $A_{s}={w\;t}_{s}$, and A_c_ is the cross-sectional area of the glass coating, defines as $A_{c}={w\;t}_{c}$.

### 4.4. Vickers Hardness

Samples (*n* = 3) for hardness testing were prepared as described in Section 4.1.1. An HM-114 Mitutoyo Testing Machine (Mitutoyo, Mississauga, ON, Canada) was utilized, equipped with a Vickers indenter, to load the samples with a force of 1 kgf (9.81 N) for 10 s. The indent diagonals were measured through the integrated optical microscope at 20×. 

### 4.5. Bulk Mode I Critical Strain Energy Release Rate Using Vickers Indentation

**Figure 8:**
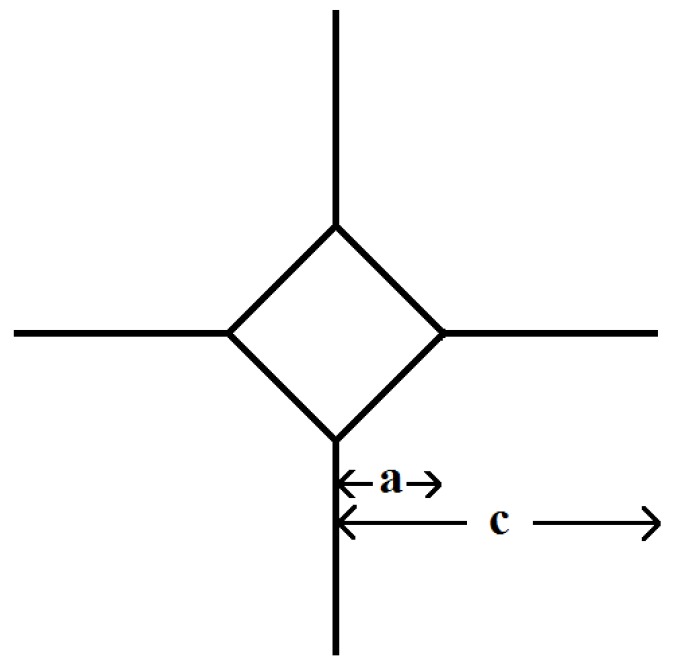
Schematic depiction of the cracks emanating from a Vickers indent. a is half of the diameter length of the dent, and c is the crack length measured from the center of the indent.

The mode I critical strain energy release rate of the bulk glasses was measured by indenting glass discs (prepared as per Section 4.1.1 and indented similarly to the process described in Section 4.4). A schematic depiction of the indentation is shown in Figure 8. According to Anstis et al. [34], the indentation load needs to be large enough to create an indent pattern that is well-defined and cracks that are longer than the indent diameter (2a), yet shorter than one tenth of the thickness of the sample (300 μm in this case) to avoid interactions with the lower free surface of the specimen. The indentation load is considered too large if it breaks the sample or causes a chipping on the sample’s surface [34].

For each glass composition, three discs were made. By trial and error, the appropriate indentation load to meet the aforementioned force criteria was found to be 3 kgf (29.43 N). This indentation load was applied using a Macro indenter HM-114 Mitutoyo Testing Machine (Mitutoyo, Mississauga, ON, Canada) normal to the surface of the glass for a duration of 10 s. The length of the crack was measured through scanning electron microscope (SEM) using a JEOL JSM-6380LV SEM (JEOL, Peabody, MA, USA). The mode I critical strain energy release rate of the bulk glass was found using the equation below [34]:

(6) $$G_{\text{IC}}=\frac{\alpha^{2}P^{2}}{{H\;c}^{3}}$$

where *P* is the applied load, *H* is hardness of the glass (as measured per Section 4.4), c is the length of the surface trace of the half penny crack measured from the center of the indent, and α is the calibration constant α=0.016±0.004. Equation (6) is derived for plain stress. Even though the current application does not completely satisfy the plain stress condition, Equation (6) can still be used as an approximation of G_IC_ given that the thickness of the discs is less than half of their diameter. 

### 4.6. Coating/Substrate System Mode I Critical Strain Energy Release Rate

**Figure 9:**
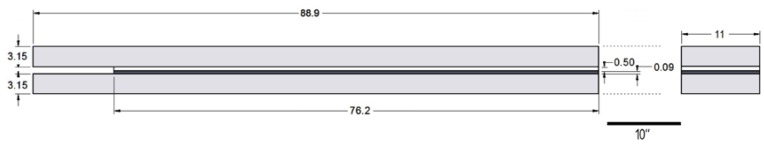
Bi-layer double cantilever beam specimens. All units are in millimeters. Gray materials represent the titanium alloy substrates, black material represents the glass, and white material represents the epoxy.

The mode I critical strain energy release rate (G_IC_) of the coating on the substrate was evaluated (*n* = 5) following the procedure outlined by Matinmanesh et al. [53]. Typical sample dimensions for the bi-layer double-cantilever beam (DCB) specimen are shown in Figure 9. Three samples per glass composition were tested. Coated samples (prepared as described in Section 4.1.2) were used to make the test specimens, then an epoxy layer (J-B Weld 8265-S Cold Weld Compound, Sulphur Springs, TX, USA) was deposited to cover the glass and attach the second titanium alloy substrate. Specimens were loaded using a STM United Tensile Tester (United Testing Systems, Inc., Huntington Beach, CA, USA) using a 500-N load cell at a rate of 0.5 mm/min; then based on the recorded loads, the mode I critical strain energy release rate G_IC_ was calculated as:

(7) $$G_{\text{IC}}=\frac{12L^{2}a^{2}}{E_{s}w^{2}t_{s}^{3}}$$

where L refers to the load to start the crack, a is the crack length, E_s_ to the tensile modulus of the substrate (110 GPa), w to the specimen width (11 mm), and t_s_ is the thickness of the substrate (3.15 mm).

### 4.7. Statistical Methods

The results of all the measurements were expressed as means with experimental scatter expressed as a standard deviation. Additionally, one-way analysis of variance (ANOVA) was employed to analyze the data to determine the significance in mean difference across the gathered data when *p* < 0.05. Post-hoc Tukey test was used on MiniTab 17 (MiniTab Inc., State College, PA, USA). The Tukey test assumes equal variance in the data sets being analyzed to determine the significance in mean difference across all factors (i.e., all glasses in both series).

## 5. Conclusions

Silica-based and borate-based glasses were synthesized and characterized in terms of their mechanical properties relevant to their use as metallic coating materials. It was observed that borate-based glasses exhibited CTE values that were closer to the substrate’s (Ti6Al4V) CTE, a common alloy used in medical implants; this translated into higher mode I critical strain energy release rates for the borate-based glasses and compressive residual stresses and strains at the coating/substrate interface, outperforming the silica-based glasses counterpart. An increase in the content of TiO_2_ in the glasses resulted in an increase in the mode I critical strain energy release rate for both the bulk glass and for the coating/substrate system. Borate-based glass BRT3, with 15 mol % TiO_2_ incorporated, exhibited superior properties overall compared to the other proposed glasses in this work, as well as Bioglass^®^ 45S5 and Pyrex.

## Figures and Tables

**Table 1:** Glass formulations (mol %).

Reagent	Silica-Based Glass			Borate-Based Glasses		
	SRT0	SRT1	SRT3	BRT0	BRT1	BRT3
SiO_2_	52	47	37	0	0	0
B_2_O_3_	0	0	0	52	47	37
CaO	12	12	12	12	12	12
P_2_O_5_	6	6	6	6	6	6
Na_2_O	14	14	14	14	14	14
ZnO	16	16	16	16	16	16
TiO_2_	0	5	15	0	5	15

**Table 2:** Glass transition, crystallization, and coating temperatures.

Glass	T_g_ (°C)	T_*x*_ (°C)	T_coat_ (°C)
SRT0	619	735	650
SRT1	592	670	630
SRT3	610	705	640
BRT0	521	603	520
BRT1	530	625	550
BRT3	523	633	550
